# Hyperspectral Fluorescence LIDAR Based on a Liquid Crystal Tunable Filter for Marine Environment Monitoring

**DOI:** 10.3390/s20020410

**Published:** 2020-01-11

**Authors:** Eleonora Aruffo, Andrea Chiuri, Federico Angelini, Florinda Artuso, Dario Cataldi, Francesco Colao, Luca Fiorani, Ivano Menicucci, Marcello Nuvoli, Marco Pistilli, Valeria Spizzichino, Antonio Palucci

**Affiliations:** Diagnostic and Metrology Laboratory. FSN-TECFIS-DIM Nuclear Fusion and Safety Technologies Department, ENEA Via Enrico Fermi 45, 00044 Frascati, Italy; andrea.chiuri@enea.it (A.C.); federico.angelini@enea.it (F.A.); florinda.artuso@enea.it (F.A.); dario.cataldi@enea.it (D.C.); francesco.colao@enea.it (F.C.); luca.fiorani@enea.it (L.F.); ivano.menicucci@enea.it (I.M.); marcello.nuvoli@enea.it (M.N.); marco.pistilli@enea.it (M.P.); valeria.spizzichino@enea.it (V.S.); antonio.palucci@enea.it (A.P.)

**Keywords:** hyperspectral, Chl-a, LIDAR

## Abstract

An innovative hyperspectral LIDAR instrument has been developed for applications in marine environment monitoring research activities, remotely detecting the fluorescence spectra produced in the spectral interval between 400 nm and 720 nm. The detection system is composed by a custom made photomultiplier charge integrating and measuring (CIM) unit, which makes automatic background signal subtraction, and a liquid crystal tunable filter (LCTF). The new instrument therefore has hyperspectral resolution and allows automatic background subtraction; it is compact and automated by custom software that permit to adapt the instrument properties depending on the environmental conditions. Laboratory tests to characterize the instrument performance have been carried out, concluding that this sensor can be employed in remote sites for Chl-a detection.

## 1. Introduction

About 71% of the Earth surface is covered by oceans and their complex ecosystems play a crucial role in climate change, absorbing about 90% of the atmospheric heat and about a quarter of the total carbon dioxide (CO_2_) emitted in the atmosphere by the fossil fuels burning [1]. The photosynthetic activity of the phytoplankton allows converting part of the absorbed CO_2_ in chromophoric dissolved organic matter (CDOM), contributing to the marine biological pump [1,2]. Moreover, phytoplankton is an important indicator of the water quality, being related to the processes of eutrophication [3]. The chlorophyll-a (Chl-a), a photosynthetic pigment contained in all the algae species, is widely used as phytoplankton proxy with in situ, remote and laboratory measurements [3,4]. The importance of monitoring the Chl-a and CDOM concentrations in marine environment is, then, extensively recognized not only to study the water quality but also to further investigate the relation between climate change and water bodies [1]. 

Instruments based on the light detection and ranging (LIDAR) technique have been widely employed for marine environment monitoring [5,6,7,8]. In marine applications, LIDAR systems adopt the Laser Induced Fluorescence (LIF) technique to remotely monitor the stimulated fluorescence, by UV laser excitation, from the dissolved or dispersed sea water components, collecting a complex continuum fluorescence spectrum. The full spectrum contains different bands that can be assigned to specific components (oils, protein-like, CDOM and phytoplankton), demanding the introduction of a dispersing element and of a digital detector (i.e., CCD). Last part of the equipment affects the final spectral resolution of the retrieved bands. Zhao et al. (2016) [5] developed an inelastic Scheimpflug LIDAR with hyperspectral resolution using a laser diode at 445 nm and a 2D array detector (2D-CCD). To have hyperspectral fluorescence signals they used a transmission grating. The instrument, which is compact and relatively inexpensive, is, therefore, capable to return up to 61 spectral bands but it is not able to totally identify the CDOM contribution, exciting with a source at 445 nm. In 2019, Duan et al. [9] constructed a drone based LIF system, employing a continuous-wave diode laser at 412 nm and a compact spectrometer to measure continuous fluorescence spectrum emitted from aquatic environment. This instrument, not employed to monitor aquatic vegetation or fauna, can represent an alternative technique to measure oil spills on water and for hydrological studies of dye dispersion. Moreover, hyperspectral LIDAR are employed with different applications to investigate the vegetation characterization [10,11], for remote sensing and mapping applied to the cultural heritage [12], for terrestrial laser scanning [13] or oil spill monitoring [14]. A LCTF-based hyperspectral LIDAR instrument with 10 nm spectral resolution and an avalanche photodiode detector has been developed to detect the vegetation red edge position [15]. 

Many LIDAR fluorosensor systems have been developed by the ENEA agency to measure Chl-a and CDOM and employed in marine, oceanographic or polar campaigns [16]. The previous configurations of the LIDAR fluorosensors allowed discriminating only discrete spectral bands because of the bandpass filters adopted in front of a battery of photomultipliers (PMTs). In fact, in these instruments the fluorescence radiation spectrum selection was demanded to a number of single bandpass filters (typically 10 nm of Full Width at Half Maximum (FWHM)), located in front of PMTs, ranging from 4 to 12. Although the high number of bandpass filters allowed a higher spectral resolution, the optical arrangement, resulted in a rail of many optical elements and PMTs, affected the compactness of the LIDAR system (in terms of weight and size). 

In this work, we present a new hyperspectral LIDAR fluorosensor. The light source is a flash lamp pumped Nd:YAG laser at 355 nm and the detection system combines a liquid crystal tunable filter (LCTF) with a custom photomultiplier (Hamamatsu model 928) charge integrating and measuring (CIM) unit patented by the ENEA agency in 2005 [17]. In this apparatus, the PMT is not used in photo-counting mode but in analog mode, where the stored current is integrated over a chosen interval of time. The system has been optimized and characterized to discriminate up to 33 spectral bands ranging between 400 nm and 720 nm with a selected resolution of 10 nm. In turn, a single electro-optical element has replaced the filters and PMTs rail, strongly reducing the overall dimensions of the apparatus. The lowest Chl-a concentration that the system has been able to discriminate during the laboratory experiments is of 0.035 µg L^−1^. The results of laboratory tests, carried out in order to characterize the CIM unit, the LCTF and the LIDAR fluorosensors, are presented in this work. In synthesis, this innovative system represents an improvement of previous configurations being a compact, potentially hyperspectral instrument, less sensitive to misalignments of the optical components. Finally, it reduces the complexity of the previous LIDAR fluorosensors systems and reduces the background interferences, allowing diurnal continuous measurements of both CDOM and Chl-a concentrations in aquatic environment. Consequently, this system can be employed in hostile environment field campaigns, as oceanographic campaigns. In spite of the advantages of this innovative technique, the use of a LCTF entails sequential acquisition of different spectral bands, which are therefore not simultaneous. In this study, however, we describe a fluorosensor LIDAR for marine applications: in this case, considering a typical cruise speed, we can assume that the environmental conditions during a complete cycle of tuning are homogeneous and then the use of the LCTF is fully justified. However, the permanence time of the LCTF employed in this study in each wavelength can be modulated and decreased up to 50 ms, allowing higher sampling frequencies. We successfully tested sampling times as low as 100 ms; however, the aim of this work did not require fast measurements.

## 2. Instrument Design

**Figure 1:**
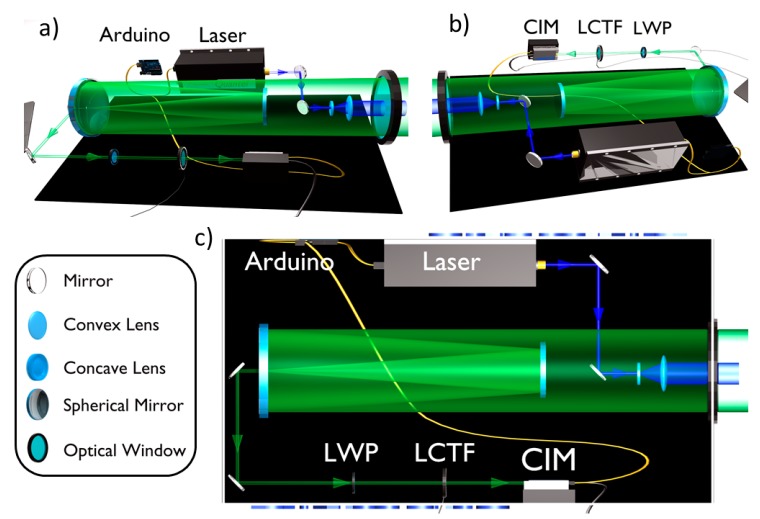
Hyperspectral LIDAR fluorosensor with its components. The excitation light emitted by the laser is sent through a Galilean beam expander to the target. The LIF signal is collected by a Cassegrain collection optics configuration and detected by a LCTF coupled with a CIM unit.

The LIDAR fluorosensor main parts are: 1) light source; 2) optics and 3) detector. Figure 1 shows schematically the instrument design with its optical layout and components. The instrument is mounted on a compact double-walled box (about 20 × 35 × 60 cm), which guarantees insulation both from solar radiation and water. The power supplies for both the laser and the remaining components are installed in independent insulated box (about 20 × 40 × 60 cm). The total weight of the LIDAR instrument is about 30 Kg. An Arduino board Uno has been conveniently programmed to trigger, by a 20 Hz TTL signal, sequentially the laser lamp, the PMT and, finally, the laser Q-switch. Pictures of the LIDAR instrument main components can be found in Appendix A.

### 2.1. LIDAR: Light Source and Optical Design

The system developed by the ENEA Diagnostic and Metrology (DIM) Laboratory is based on a flash lamp pumped frequency tripled Nd:YAG laser (model Quantel Ultra 100) emitting at 355 nm, with a maximum of 20 Hz of repetition rate, up to 30 mJ of pulse energy and 8 ns of pulse duration (Figure 1). A Galilean 5x beam expander is implemented in order to reduce the laser divergence to 1 mrad and the energy density at the target. The beam diameter at the exit of the instrument is 2.5 cm. This makes the instrument less affected by problems related to accidental exposure of skin or eye to the beam or its reflection on water. In the UVA region, the maximum permitted exposure does not depend on the beam divergence (IEC 60825-1 International Standard and European Directive 2006/25/EC). Further details about the eye-safe laser systems can be found elsewhere [18].

The collection optics is realized with a Cassegrain configuration, employing a 150 mm primary spherical mirror (Figure 1). This allows large focal length into a compact design, with a relatively large F number (F/6.7). A field stop is placed in the focal plane to adjust the field of view (FOV) and limit the background illumination, and after the stop a lens collimates the beam. Then, a longpass dichroic mirror Semrock BLP01-355R-25 (long wave pass (LWP) in Figure 1) is implemented to remove most of the elastic laser echo (OD = 7@355 nm, T = 97% between 370 and 700 nm) before entering the LCTF (Cri’s VariSpec model VIS-10-20, 375 gr of weight, 8.5 × 5 × 5 cm, USB type mini-B serial control). The FOV of the system is given by the ratio between the stop diameter and the focal length. With the stop adjustable between 1 and 8 mm over a focal length of 1000 mm, the FOV may span between 1 and 8 mrad. In any case, this value is adjusted to frame the laser spot in order to discard the background noise from the non-illuminated region of the target. The telescope can be focused to a distance dependent on the installation layout on board the ship. In fact, this distance can span from 5 to 15 m depending on the ship and the deck, and it is usually chosen so that the maximum of the optical efficiency falls about 2 m underwater. Indeed, neglecting the spherical aberrations of the mirrors, our calculations show a depth of focus (DOF) of about 6 m FWHM if the telescope is focused 15 m away from the target [19]. This means that the data are averaged with a bell-shaped weighting function over the first 5 m of sea water [19]. However, the total weighting function also contains a term related to the extinction of both the laser beam and the fluorescence echo, a term quite difficult to determine since it depends on the water turbidity and depth. Moreover, it is well known that the extinction in water strongly depends on the wavelength, so the weighting functions are different for each band. As a result, the exact scattering volume is actually unknown. However, for this reason one of the channels records Raman echoes from liquid water at around 405 nm. Since the Raman signal from sea does not depend on the sample concentration, it just gets modulated by sea-instrument distance, laser power fluctuations and overall optical efficiency. All of these terms affect the signal regardless of the wavelength and may be cancelled out by normalizing the fluorescence signal to the Raman one, with the only exception of sea water differential extinction, which may be quite different from blue to red and some uncertainty on the scattering volume still remains [20]. Nevertheless, in general the measurements can be considered as representative of the first few meters of water.

### 2.2. Detector

An LCTF, coupled with a photomultiplier integrated in the CIM unit [17], forms the detection system of the fluorescence signal (see Figure 1 for a schematic illustration of both these components).

**Figure 2:**
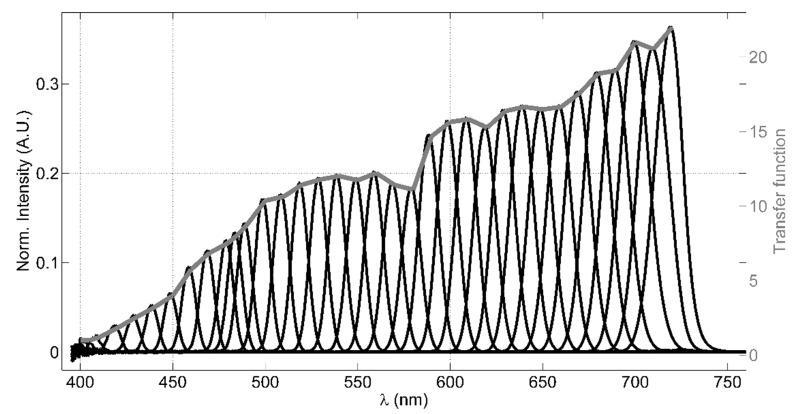
The LCTF transfer test results: (1) the black lines represent the intensities measured at each tuned wavelength by the spectrometer normalized by the light source spectrum; (2) the grey line is the evaluated transfer function.

The LCTF filter is a solid-state tunable birefringent filter, with electronically controlled liquid crystal elements that allows to select a specific working wavelength. The visible-wavelength model, employed in the LIDAR instrument, has an operational wavelength range between 400 nm and 720 nm, with bandwidths (FWHM) of 10 nm, 22 mm of clear aperture and an optics response time of 50 ms. A value of 4 is declared as OD of the out-of-band spectral regions; this allows high rejection of spurious signals, apart from the laser elastic echo that is cut by means of the longpass dichroic beamsplitter. A custom software (based on LabView) allows to set a wavelength palette, in which the filter has to be cyclically tuned, and the residence time of the LCTF in each tuned wavelength. This configuration allows acquiring spectrally resolved fluorescence of the signal originating from the target under analysis, adapting both the tunable wavelengths and the residence time of the LCTF in each wavelength depending on the experimental conditions. Considering that the VariSpec’s LCTF transfer function is wavelength-dependent, a laboratory test for measuring this parameter is necessary in order to evaluate an appropriate transfer function to be applied to the transmitted intensity registered by the photomultiplier. Figure 2 shows the measured transmittance of the LCTF as function of the wavelength, obtained using a filament tungsten lamp as a light source and a compact QE-Pro Ocean Optics spectrometer (operating in the range 250–947.5 nm with a bandwidth of 2.5 nm) as the detector, which collects the light by a fiber optic. The intensity (arbitrary unit), normalized by the lamp spectrum measured in case the LCTF is not placed between the lamp and the fiber optic, is shown (black lines) in Figure 2. 

The transfer function, then, has been evaluated dividing the normalized intensity of each wavelength by the signal measured at 400 nm (grey line in Figure 2). In this test, we choose to tune the LCTF to 35 wavelengths ranging between 400 nm and 720 nm, typically with a step of 10 nm between them, residing 60 s in each wavelength and averaging three spectra integrated for 100 ms. Since the typical bandwidth FWHM is 10 nm, acquiring signals with a 10 nm step provides a well-resolved spectral reconstruction in the VIS range. A simple model has been used to verify the effect of the LCTF transfer function (see Appendix B for more details). Despite the LCTF transmittance is strongly dependent on the light polarization, we did not take into account this effect in our system considering that the fluorescence signal is not polarized.

The signal acquisition module selected for the LIDAR instrument is a charge integrating and measuring unit [17], previously integrated in ENEA’s LIDAR systems. The unit allows not only to control the photomultiplier, but also to automatically remove the background contribution jointly to the use of a reference light emitting diode (LED). The total number of photons emitted by the target in the telescope field of view reaching the PMT (*Q_PMT_*), neglecting the dark current term, is given by:

(1) $$Q_{PMT}=\int_{\Delta T}I_{BGND}(t)dt+\int_{\Delta T}I_{LIF}(t)dt{=}_{}Q_{BGND}+Q_{LIF}$$

where $I_{LIF}\left(t\right)$ and $I_{BGND}\left(t\right)$ are the contributions to the current signal at the PMT anode from the pulsed LIF signal and the diffuse sun light, respectively. It is possible to subtract the background contribution from the LIF signal applying a time discrimination method and dividing the *Q_PMT_* signal in sub-intervals in which only the background or both the background and the LIF signals occur. 

Typically, the background cancellation procedure requires consecutive measurements with and without the LIF contribution. To reduce the dynamic range that this approach requires, a smart time discrimination method has been implemented. Defining two paths to integrate the anode current signals, the equation describing the *Q_PMT_* becomes:

(2) $$Q_{PMT}=-\frac{1}{m}\int_{t_{0}}^{t_{0}+t_{1}}I_{PMT}\left(t\right)dt+\int_{t_{0}+t_{1}}^{t_{0}+t_{1}+\Delta T_{1}}I_{PMT}\left(t\right)dt-\frac{1}{m}\int_{t_{0}+t_{1}+\Delta T_{1}}^{t_{0}+\Delta T_{2}}I_{PMT}\left(t\right)dt$$

(3) $$Q_{PMT}=-\left(B_{0}+\frac{1}{2}B_{1}\Delta T_{2}\right)\frac{\Delta T_{2}-\Delta T_{1}\left(m+1\right)}{m}+\;Q_{LIF}$$

where t_0_ is the start time of gate G1, *m* is a constant term ($m=\Delta T_{1}/\Delta T_{2}$), $t_{0}+t_{1}$ is the initial time of G2 gate (which contains the LIF signal). Integrating Equation (2), with the assumption that the background signal B_1_ has a nearly constant amplitude during $\Delta T_{2}$, we obtain *Q_PMT_* as in Equation (3). It is worth noticing that the choice made for *m* makes the term inside the square bracket of Equation (3) vanish, so that the background has null net contribution regardless from the background amplitude. This new proposed method allows that the smart gate and gain definition remove automatically the background contributions from the LIF signal measurement, i.e., $Q_{PMT}\approx Q_{LIF}$. As a consequence, a suitable selection of temporal intervals allows to automatically remove the background contributions simultaneously to the LIF signal measurement.

**Figure 3:**
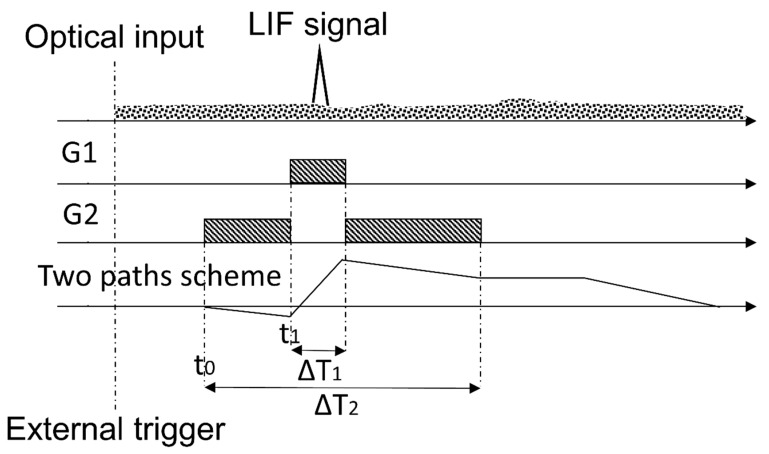
Gates scheme and their relations, as implemented in the CIM unit. In figure, the shaded rectangles represent the temporal intervals when each gates G1 and G2 are active; the triangle schematizes the LIF signal and the dotted area on the top is a schematic illustration of the background signals. The continuous line in the last subplot represents the optical input (first plot) integrated with negative gain -1⁄m in the time interval G2 and with unity gain in the time interval G1.

To solve Equation (2), the definition of temporal intervals (henceforth, gates) accurately related among them is necessary. Figure 3 represents schematically the gates scheme defined in the CIM unit: G1 is the temporal interval in which the LIF signal (plus the background) is sampled and G2 is the temporal interval in which the background signal is sampled. In synthesis, as the external trigger is activated, the PMT gain is switched on. After a defined interval of time, the storage capacitor starts the integration with initial gain set to −1/m, then to +1 and, finally, again to −1/m (as described by Equation (2) and illustrated in Figure 3). An additional total delay, which can be either a positive or a negative term, is defined in order to shift the common start of the gates: this allows to adapt the delay between the PMT gain switching on and the arrival of the LIF signal. Further information can be found elsewhere [17]. By using the CIM unit system with an automatic background subtraction, the measurements do not depend on the environmental illumination conditions. Moreover, this automatic procedure is done continuously and simultaneously with the fluorescence signal measurements: therefore, it is not necessary to interrupt the sampling to evaluate periodically the background as required by other methodologies. Moreover, it is important to observe that both the gain of the PMT and the integration time can be modulated by changing the dynode chain voltage. Consequently, depending on the measurement conditions, it is possible to avoid the PMT saturation, decreasing the gain and/or the integration time, or to improve the instrument sensitivity up to its detection limit. Laboratory tests on the sensitivity of the CIM unit are presented in the following paragraphs. 

**Figure 4:**
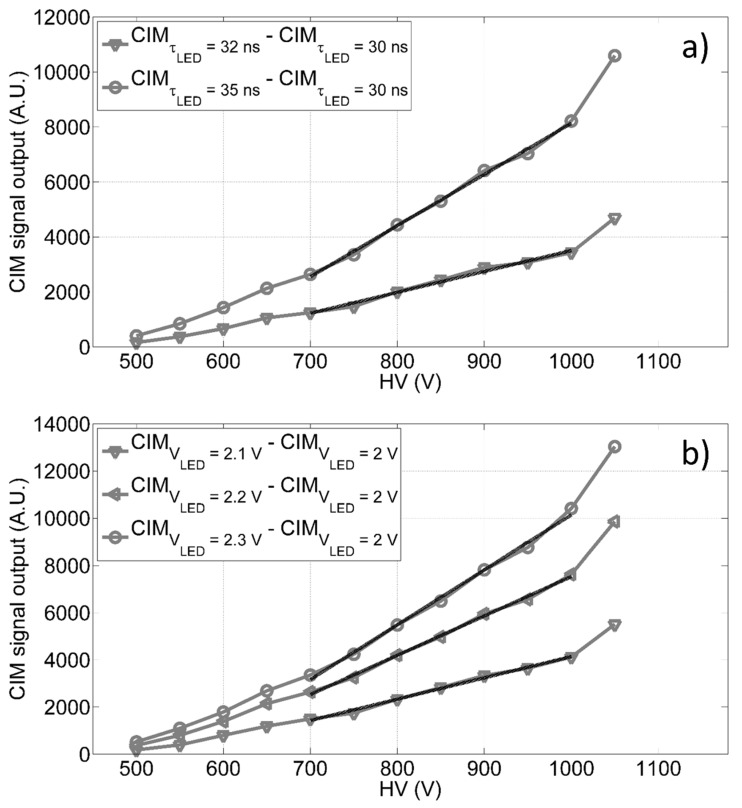
CIM differential output as function of the anode to cathode voltage in diverse conditions: (**a**) difference between the CIM output measured at 35 ns and 32 ns of pulse widths and the reference signal measured at 30 ns with constant pulse amplitude; (**b**) difference between the CIM output at 2.1 V, 2.2 V and 2.3 V of LED pulse amplitudes and the reference signal observed at 2.0 V with constant pulse width.

The CIM unit has been characterized by laboratory tests, using different configurations of light pulses generated from a set of LEDs, mounted on a cylinder support fixed on a stackable lens tube (with an internal diameter of 2.5 cm), which is part of the CIM unit and installed on correspondence of the PMT window. Up to five LEDs have been run independently by a custom power supply module, allowing to set the working conditions of LEDs, i.e., amplitude, pulse width and frequency. Tests have been carried out to verify the trend of the CIM unit with respect to the gain and in different environmental conditions, evaluating its signal amplitude as a function of: A) different LED signal pulse widths, and B) different LED signal pulse amplitudes. We measured the receiving unit signal outputs for given light intensity (i.e., reference level, 30 ns of pulse width and 2 V of pulse amplitude) and, then, we evaluated the incremental trends for different signal level amplitudes (or widths) with respect to the relative reference (as shown in Figure 4). The findings in Figure 4 show a linear trend (see Table 1) of the CIM unit signal output versus the bias voltage in the range 700 V to 1000 V, thus giving us the opportunity to dynamically adapt the PMT gain, and consequently the receiving unit spectral sensitivity, to the experimental conditions. 

**Figure A4:**
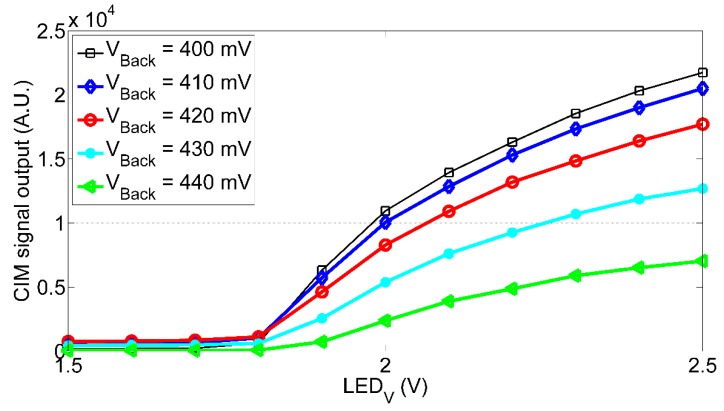
CIM signal output as function of the LED signal pulse amplitude for different background levels.

The sensitivity of the CIM unit has been analyzed in different background conditions, studying its signal output as a function of the variable LED light pulse. The background variation has been simulated using a second continuous LED supplied with different current levels. We found that, as the background increases, the sensitivity of the CIM unit decreases (shown in Figure A4). Finally, the differential linearity of the CIM unit in several environmental conditions has been evaluated by laboratory tests based on the finite-difference method [21]. The results are described in Appendix D. The CIM unit has been successfully employed in previous configurations of LIDAR fluorosensor built by the ENEA and operated in many Arctic and Antarctic campaigns to monitor, for example, CDOM and Chl-a concentrations off Svalbard [22] and oil spill in the Mediterranean Sea [23].

## 3. Laboratory Tests and Discussion

Laboratory tests have been carried out in order to characterize the LIDAR fluorosensor performance, focusing on the Chlorophyll-a detection.

### 3.1. Experimental Setup

**Figure 5:**
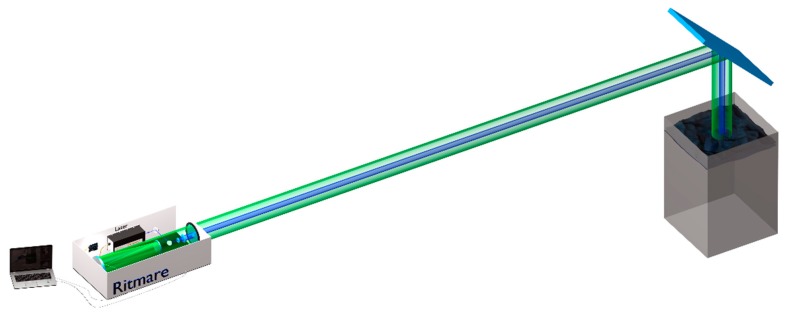
Experimental setup: the target has been located 15 m far from the LIDAR system, considering the telescope focusing setup (described in Section 2.1).

The newly developed sensor configuration can be adapted to different open water basins or seawater environments. In view of a forthcoming marine measurement campaign, the LIDAR system has been focused for a target distance of about 15 m. An aluminated mirror at 45 deg has been used to deflect the laser beam towards a polyethylene cylinder (~45 cm height, ~35 cm diameter) containing 40 L of double-distillated water (Merck Millipore Elix). At the bottom of the cylinder, we placed a matte aluminum foil to eliminate the interferences originated by the cylinders bottom surface (see Figure 5 for a schematic illustration of the experimental setup). The use of a matte aluminium foil leads to the diffusion of the laser beam reaching the bottom of the cylinder that could excite fluorescence from the plastic sides of the cylinder; anyway, an exact calibration was beyond the aim of this study. The total delay between the PMT gain switching on and the LIF signal has been evaluated and set to 4.2 ns. We set the laser with a repetition rate of 20 Hz. The data stored were the result of the average on the signal acquired over 20 laser pulses. The anode to cathode high voltage supply varied between 700 V and 1100 V, depending on the specific experiment and instrument optimization requirements. Finally, the LCTF has been cyclically tuned on 33 wavelengths between 410 nm and 720 nm, with interval of 10 nm, and an additional acquisition at 405 nm; the residence time on each wavelength was 5 s (i.e., five stored data per wavelength at each tuning cycle) for a total measurement time per spectrum of 160 s.

Mixtures with known amount of Chl-a in different concentrations have been used to test the sensitivity of the LIDAR. The reference mixture containing a known amount of Chl-a has been prepared through extraction of bay leaves using 60 mL of 100% acetone. The leaves were allowed to soak for 5 min. The extract has been filtered by a coarse funnel with a paper filter and further clarified with a 0.2 μm nylon filter syringe. The pigment content of the extract has been characterized and quantified through High Pressure Liquid Chromatography (HPLC) analysis in our laboratory facility. The HPLC method used in this work is a modified version of the protocol developed by Wright et al. (1991) [24]. The extract was injected through a 100 μL loop into the HPLC Agilent Technologies system (quat. pump 1260 VL, diode array detector 1260 DAD VL) equipped with a Supelco Ascentis C18 column (25 cm, 4.6 mm ID, 5 μm size). The elution was performed at a flow rate of 1 mL min^−1^ using a linear ternary gradient with detection set at 450 nm and 436 nm. Identification and quantification of pigments was performed using certified standard solutions purchased from DHI Water and Environment Institute (Denmark). The concentration of Chl-a in the solution has been found to be 690 µg L^−1^. The mixture also contained the following pigments: neoxathin (20 µg L^−1^) violaxanthin (20 µg L^−1^), lutein (80 µg L^−1^), Chl-*b* (220 µg L^−1^), α-carotene (20 µg L^−1^), β-carotene (60 µg L^−1^). The final Chl-a concentrations, obtained by sequential dilution in the 40 L of double-distilled water, in the mixture employed during the laboratory tests varied between 0.007 µg L^−1^ and 1 µg L^−1^.

### 3.2. Discussion about Water Raman and Chl-a Signals

**Figure 6:**
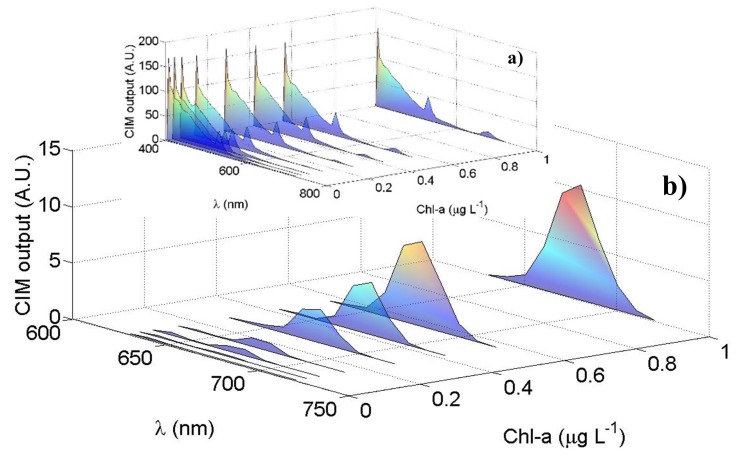
Laboratory test results: the PMT mean output signal (A.U.) as function of the wavelength (nm) for different Chl-a concentrations (µg L^−1^): (**a**) the overall spectra between 405 nm and 720 nm; (**b**) the Chl-a peaks highlighted in the spectral region between 640 nm and 720 nm with their maxima at 680 nm.

Figure 6 shows the results obtained by laboratory tests using different and known concentrations of Chl-a. The data shown are corrected by the LCTF transfer function (see Section 2.2) and by the PMT quantum efficiency, which can be approximated by the following function, referencing to the PMT datasheet employed in this instrument: $PMT_{quantum\;efficiency}=146.5e^{-0.0046\lambda }$. An overall spectral transfer function of the LIDAR cannot be easily and accurately performed by considering all the single optical components (windows, mirrors, filters and beamsplitters…) also taking into account that their properties can change over time. The transfer function, in fact, is typically retrieved by a calibration curve obtained by comparison of the signal from a reference sample by a reference instrument, such as a benchtop spectrophotometer. Moreover, if the instrument is requested to provide concentration of specific compounds (as for example chlorophyll, CDOM …) another procedure is usually adopted. In this case, in fact, the calibration is performed after the analysis of significant data from the spectra (i.e., ratios between specific band), through the comparison of concentrations obtained by reference techniques. In Figure 6a we observe that the water Raman signal at 405 nm as well as the signal between 410 nm and 650 nm remain constant in case of varying the Chl-a concentrations. In Figure 6b the averaged Chl-a peaks at 680 nm, as function of the Chl-a concentrations, are highlighted: as expected, the measured peak at 680 nm increases as the Chl-a concentration in the 40 L cylinder increases from 0.007 µg L^−1^ up to 1 µg L^−1^. Figure 6a reports a residual contribution of the 532 nm harmonic of the laser source is present in the measured spectra (see also Appendix B).

**Figure 7:**
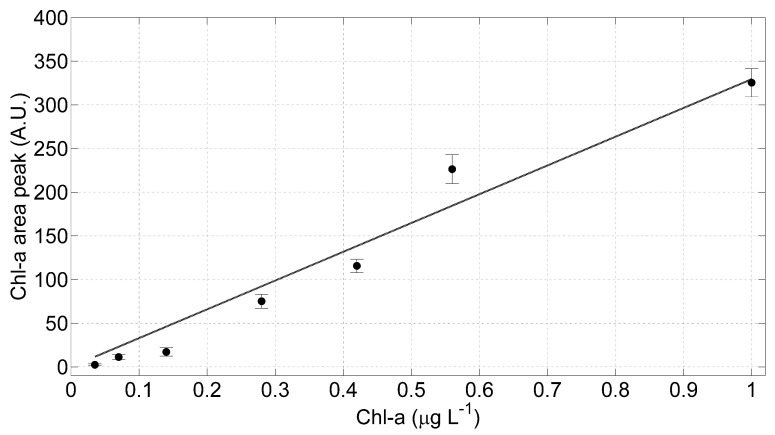
The LIDAR fluorosensor linearity test results. Linear regression model (grey line) has been applied to the measured data corresponding to the Chl-a mean curves and the relative standard deviation.

To verify the linearity of the optical sensor, the areas under the mean curve in Figure 6b have been numerically calculated and plotted as a function of the Chl-a concentrations (Figure 7); a linear regression model has been applied. The error bars in Figure 7 have been evaluated as the maximum variation of the fitted areas within the samples acquired for each wavelength. Applying linear regression models to these data and evaluating the t-Student critical values (t), we found that the intercept is not significantly different from zero: the calculated t, in fact, is lower than the corresponding one found in the tabulate t-distribution with a significance level of 0.05. Consequently, we applied to the measured data a linear regression model with null intercept: the estimated coefficients of this model with relative standard error (SE) and the statistical parameters employed to estimate the goodness of the evaluated fit are listed in Table 2.

Moreover, we applied the Hartley test to analyze the variance homogeneity: the calculated Hartley F_MAX_ is ~291 and the tabulate is ~333 for a significance level of 0.05. It is possible, then, to consider the data variance homogenous: it follows that the obtained parameters with the RMS method are not affected by different fluctuations among the considered samples. In conclusion, we found a statistically significant linearity in the LIDAR fluorosensor signals increasing the Chl-a concentrations on the samples under analysis. Finally, the lowest Chl-a concentration that the LIDAR fluorosensor has been able to measure during these laboratory tests is of about 0.035 µg L^−1^, a value below the range of the Chl-a level registered in background site, such as Artic or Antarctic marine environment [16].

## 4. Conclusions

The study of the phytoplankton and the dissolved organic matter in aquatic environments and, especially, in oceanographic campaigns is of crucial importance not only to investigate the climate change and its interaction with water bodies, but also to monitor water pollutions [1]. At the Diagnostics and metrology Laboratory (FSN-TECFIS-DIM) of the ENEA agency, LIDAR fluorosensors to monitor aquatic environments have been developed since 1999. Recently, a new hyperspectral LIDAR fluorosensor has been realized introducing a new electro-optical elements acting as a single hyperspectral dispersing device. This instrument allows remote, continuous and real time measurements of both CDOM and natural occurring phytoplanktonic community measurements with automatic background subtraction and 33 spectral bands discrimination. The CIM unit used in this instrument shows linearity in different measurements conditions; moreover, the possibility to vary the cathode gain of the PMT between 700 V and 1000 V allows the employment of the LIDAR fluorosensors in different environments avoiding the system saturation and increasing its sensitivity, if necessary. As consequence, the instrument, which is compact, automated and controlled by custom software and with a linear response with respect to the Chl-a concentrations, can be in real time adapted for different exigencies depending on the laboratory or field campaigns conditions. The implementation of the new LCTF filter, then, reduced the system complexity also in terms of control and acquisition electronics. The lowest Chl-a concentration detected during the laboratory test is of 0.035 µg L^−1^ and permits to use this system in oceanographic campaigns in remote sites, such as polar campaigns.

## Figures and Tables

**Table 1:** The goodness of fitness (GOF) parameters for the linear trends shown in Figure 4 (evaluated between 700 V and 1000 V): R^2^ is the R-squared and RMSE is the root mean squared error.

Curve	Fit Coefficients(y=p1*x+p2, with 95% Confidence Bounds)	R^2^	RMSE
$CIM_{\tau_{LED=32\;ns}}$-$CIM_{\tau_{LED=30\;ns}}$	p1 = 7.6 (6.6, 8.6) p2 = −4100 (−4967, −3232)	0.987	104
$CIM_{\tau_{LED=35\;ns}}$-$CIM_{\tau_{LED=30\;ns}}$	p1 = 18.6 (17.4, 19.9) p2 = −1.048 × 10^4^ (−1.156 × 10^4^, −9403)	0.997	1230
$CIM_{V_{LED=2.1\;V}}$-$CIM_{V_{LED=2\;V}}$	p1 = 9.0 (8.2, 9.9) p2 = −4905 (−5631, −4178)	0.993	87
$CIM_{V_{LED=2.2\;V}}$-$CIM_{V_{LED=2\;V}}$	p1 = 16.7 (15.6, 17.8) p2 = −9145 (−1.006 × 10^4^, −8226)	0.997	111
$CIM_{V_{LED=2.3\;V}}$-$CIM_{V_{LED=2\;V}}$	p1 = 23.2 (21.4, 25.1) p2 = −1.3 × 10^4^ (−1.5 × 10^4^, −1.1 × 10^4^)	0.995	195

**Table 2:** Goodness of fit analysis. The linear regression model parameters and relative statistical indexes.

	Estimated Slope	SE	tStat	*p*-Value	RMSE	R-Squared
**Chl-a Peak Area**	329.4	19.5	16.9	2.8 × 10^−6^	24.6	0.97

**Figure A1:**
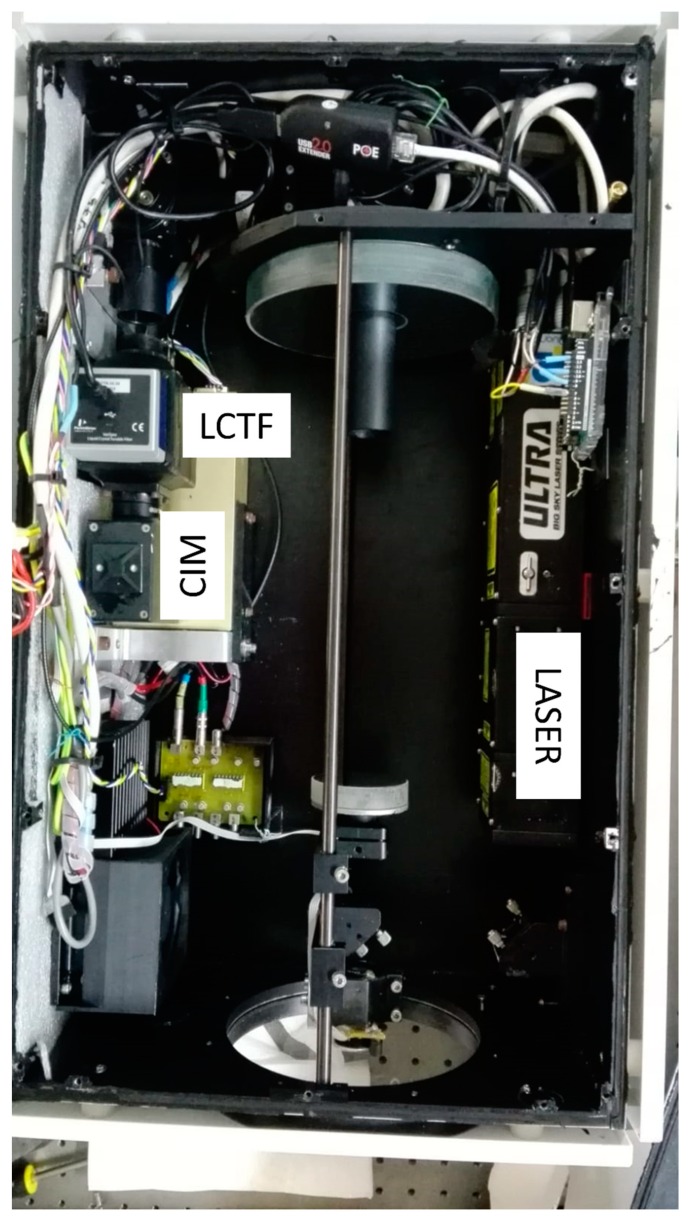
Photo of the LIDAR instrument main box: details of the laser, optics and detector.

**Figure A2:**
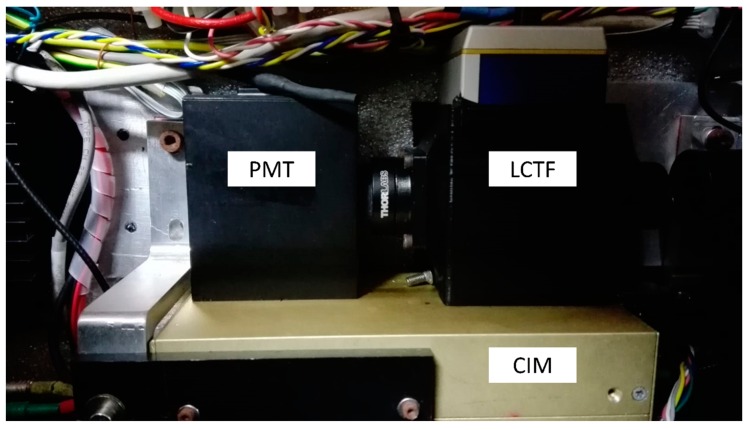
Photo of the CIM unit with the LCTF apparatus mounted on his top.

**Figure A3:**
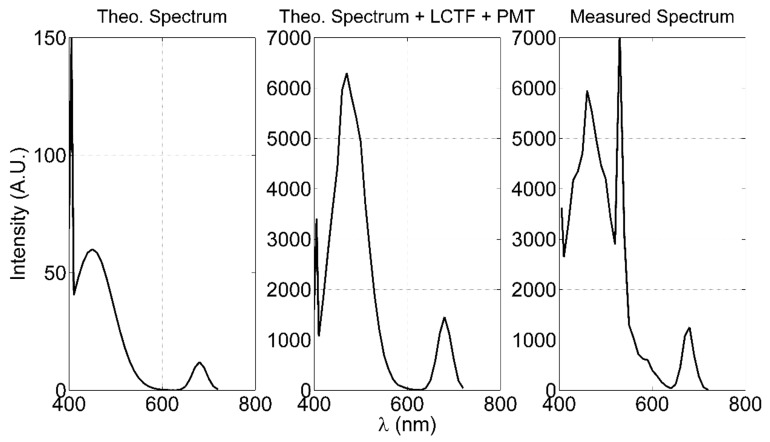
LCTF transfer function test. From the left: the theoretical fluorescence water spectrum; the convolution between the theoretical spectrum and the LCTF transfer function, as shown in Figure 2 of the main text.

**Figure A5:**
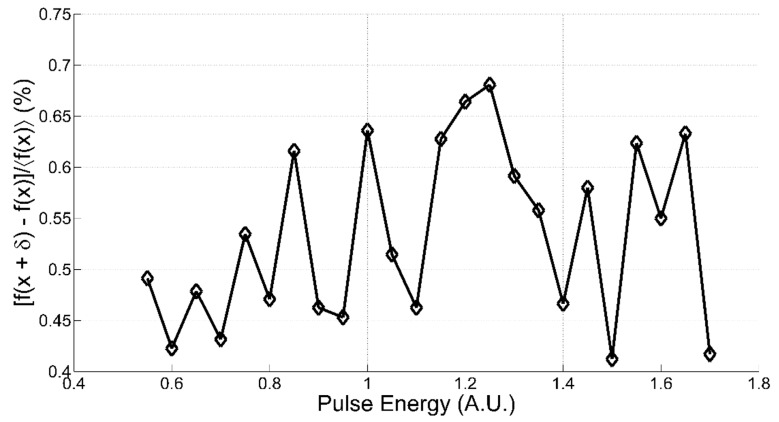
The finite-difference response curve as function of the variable LED supply voltage (anode to cathode voltage levels of the PMT at 900 V).

## Appendix A. CIM and LIDAR Photos

Figure A1 and Figure A2 shows photos of the LIDAR instrument main box and a detail of the CIM unit and the LCTF detector, respectively.

## Appendix B. Further Test on the LCTF: Transfer Function Characterization

To verify the effect of the LCTF transfer function on the acquired spectra, we evaluated a theoretical spectrum of the water fluorescence assuming that it can be composed by the sum of three contributions peaked at 405 nm (water Raman peak), 450 nm (CDOM peak) and 680 nm (Chl-a peak), respectively (first panel in Figure A3). Then, the convolution between the modelled spectrum and the LCTF transfer function curve (second panel in Figure A3) corrected by the PMT quantum efficiency has been calculated and compared with an uncorrected spectrum acquired by the LIDAR fluorosensor (third panel in Figure A3) during the laboratory tests described in Section 3. It is evident that the LCTF transmittance allows to detect the typical features on the water fluorescence and Raman spectrum. In the measured spectrum a peak at 532 nm shows up: it is a residual contribution of the 532 harmonic of the laser source.

## Appendix C. CIM Sensitivity in Different Background Conditions

The sensitivity of the CIM unit has been investigated by considering different background conditions. As described in Section 2.2 a pulsed (continuous) LED simulated the signal (background). The results are shown in Figure A4. We varied the amplitude of the background LED between 400 mV and 440 mV: the CIM signal output decreases as the background level increases.

## Appendix D. CIM Sensitivity in Different Background Conditions

The differential linearity of the CIM unit in different environmental conditions has been tested. For this purpose, we fed two independent LEDs: one with variable current level (“variable” LED) and the other with fixed and lower pulsed current (δ LED). We then measured the CIM response function flushing only the variable LED (f(x)) and flushing simultaneously both the LEDs (f(x+δ)). The difference f(x+δ)-f(x) is a measure of the CIM unit differential linearity [21]. We obtained the results in Figure A5, which shows a linear behavior of the system with a fluctuation of about 0.3% at 900 V of cathode to anode voltage levels.
